# HIV-1 drug resistance genotyping from antiretroviral therapy (ART) naïve and first-line treatment failures in Djiboutian patients

**DOI:** 10.1186/1746-1596-7-138

**Published:** 2012-10-08

**Authors:** Aden Elmi Abar, Asma Jlizi, Houssein Youssouf Darar, MohamedAliBenHadj Kacem, Amine Slim

**Affiliations:** 1 Laboratoire de la Caisse Nationale de Sécurité Sociale, Djibouti, Republic of Djibouti; 2 Laboratoire de Microbiologie, CHU Charles Nicolle, Tunis, Tunisie; 3 Service des Maladies Infectieuses de l’Hôpital Général Peltier, Djibouti, Republic of Djibouti

**Keywords:** HIV-1 drug resistance, First-line antiretroviral therapy failure, Antiretroviral naive, Djibouti, Virological failure

## Abstract

**Abstract:**

In this study we report the prevalence of antiretroviral drug resistant HIV-1 genotypes of virus isolated from Djiboutian patients who failed first-line antiretroviral therapy (ART) and from ART naïve patients.

**Patients and methods:**

A total of 35 blood samples from 16 patients who showed first-line ART failure (>1000 viral genome copies/ml) and 19 ART-naïve patients were collected in Djibouti from October 2009 to December 2009. Both the protease (PR) and reverse transcriptase (RT) genes were amplified and sequenced using National Agency for AIDS Research (ANRS) protocols. The Stanford HIV database algorithm was used for interpretation of resistance data and genotyping.

**Results:**

Among the 16 patients with first-line ART failure, nine (56.2%) showed reverse transcriptase inhibitor-resistant HIV-1 strains: two (12.5%) were resistant to nucleoside (NRTI), one (6.25%) to non-nucleoside (NNRTI) reverse transcriptase inhibitors, and six (37.5%) to both. Analysis of the DNA sequencing data indicated that the most common mutations conferring drug resistance were M184V (38%) for NRTI and K103N (25%) for NNRTI. Only NRTI primary mutations K101Q, K103N and the PI minor mutation L10V were found in ART naïve individuals. No protease inhibitor resistant strains were detected. In our study, we found no detectable resistance in ∼ 44% of all patients who experienced therapeutic failure which was explained by low compliance, co-infection with tuberculosis and malnutrition. Genotyping revealed that 65.7% of samples were infected with subtype C, 20% with CRF02_AG, 8.5% with B, 2.9% with CRF02_AG/C and 2.9% with K/C.

**Conclusion:**

The results of this first study about drug resistance mutations in first-line ART failures show the importance of performing drug resistance mutation test which guides the choice of a second-line regimen. This will improve the management of HIV-infected Djiboutian patients.

**Virtual slides:**

The virtual slide(s) for this article can be found here: http://www.diagnosticpathology.diagnomx.eu/vs/2051206212753973

## Background

Since the discovery of human immunodeficiency virus (HIV) as the causative agent in 1983, the HIV-1 pandemic is characterized by an extensive variability and the circulation of numerous genotypes and recombinant forms (CRFs) [1,2]. Subtype C is the most prevalent, estimated to represent 48% infections in the HIV-1 pandemic [3]. Subtype C is predominant particularly in south and east Africa [4]. This strongly contrasts with the situation in central Africa where a marked genetic heterogeneity is observed.

HIV-1 remains a crucial global health problem. Delivery of ART in low and middle income countries has required utilization of a public health approach in which standardized, rather than individualized, regimens are prescribed to very large numbers of HIV-1-infected individuals [5]. At present, the majority of individuals in these countries are initiating first-line therapy with a non-nucleoside reverse transcriptase inhibitor (NNRTI) and two nucleoside reverse transcriptase inhibitors (NRTI) [6].

The introduction of antiretroviral (ARV) therapy in resource-poor settings is effective in suppressing HIV-1 replication and prolonging life of infected individuals. Nevertheless, patients in routine clinical care experience sustain detectable viral replication even under potent combination therapy. Various factors underlie this failure to achieve viral suppression, but resistance to current medications has been increasingly recognized as an important factor. Rational choice of the NRTI component of second-line therapy should be based on patterns of resistance developed during first-line therapy [7].

Djibouti is the smallest country in east Africa. The estimated prevalence of HIV-1 infection was around 2.5% (2009). Since the introduction of antiretroviral therapy in 2004, no data exist on the epidemiology of drug resistance mutations and little was known about HIV-1 genotypes circulating in Djibouti. Data on drug resistance in the routine care setting are urgently required to check ART efficacy. The present study aims to characterize circulating HIV-1 subtypes and to identify drug resistance mutations in patients who first-line therapy failed (>1000 copies/mL) and antiretroviral-naive patients from Djibouti.

## Materials and methods

### Population study and sample collection

Between October and December 2009, the whole blood samples were collected in Djibouti city from 35 HIV-1 infected patients consulting at three health centers (Hôpital General Peltier, N = 23, Centre Yonis Thoussaint, N = 9 and Centre Paul Faure, N = 3). EDTA (ethylenediaminetetraacetic acid-anticoagulated) plasma specimens were obtained after centrifugation, and stored at −80°C in aliquots for later assessment of HIV RNA viral-load and genotypic drug resistance testing. The ethical aspects of this study were approved by the local committee. For all participants, blood collection was performed after informed consent, under the supervision of local sanitary authorities.

### Viral load and CD4

The CD3/CD4+ cells were enumerated by flowcytometry (BD FACSCount System) and the quantification of HIV-1 viral load (VL) was measured using NASBA, NucliSENS®EasyQ Assays (BioMerieux) at the central laboratory of “Hôpital General Peltier” in Djibouti.

The plasma samples were shipped to the Laboratory of Microbiology of “Charles Nicolle” Hospital (Tunis, Tunisia) for drug resistance genotyping tests.

### Drug resistance genotyping

For Drug Resistance genotyping, the viral RNA was isolated from plasma samples using DSP virus RNA kit (QIAGEN Inc.) and reverse transcribed to make complementary DNA (cDNA) copies using a cDNA synthesis kit the One Step RT-PCR (QIAGEN Inc). The reverse transcriptase (RT) and protease (PR) genes were amplified according to the ANRS consensus sets of primers: for the RT gene amplification, MJ3 and MJ4 primers were used for a first PCR (RT-PCR), and A(35) with NE1(35) as inner primers for nested PCR. For the protease gene amplification, 5′Prot1 and 3′Prot1 outer primers were used and the 5′Prot2 and 3′Prot2 as inner primers: (ANRS: http://www.hivfrenchresistance.org/ANRS-procedures.pdf).

RT-PCR involved a reverse transcription step at 50°C for 30 minutes, followed by 95°C for 15 minutes before proceeding to 40 cycles of 95°C for 45 seconds, 50°C for 30 seconds and 72°C for 1 minute 30 seconds, with a final extension step of 10 minutes at 72°C. The RT-PCR products were then subjected to nested PCR with the following cycling conditions: a denaturation step of 2 minutes at 95°C, and then 35 cycles with 1 cycle consisting of 30 seconds at 95°C, 30 seconds at 55°C and 30 seconds at 72°C with a final extension at 72°C for 7 minutes.

PCR products (5 μL) were resolved by electrophoresis on a 1.5% agarose gel *GellyPhorLE, (Euroclone, UK)*. DNA bands were stained with *RedSafe™ Nucleid Acid Staining Solution [20,000*_*X*_*] (iNtRON Biotechnology, Inc, Korea)*. They were then visualized in UV light to assess the presence of fragments of the expected length compared with the standard marker *SharpMass™50-DNA ladder (Euroclone, Wetherby, UK)*. The amplification products were purified using QIAquick PCR Purification kit (QIAGEN Inc).

The PCR products were finally sequenced on an ABI3130 Genetic Analyser (Applied Biosystems), and compared with HXB-2 sequence using SeqScape v2.6 software (Life technologies). All sequences were subjected to quality assessment and determination of DR mutation profile (susceptible, intermediate resistance and resistance) using the Stanford Database (available at http://hivdb.stanford.edu/).

## Results

This study includes 35 HIV-1-infected Djiboutian patients enrolled between October and December 2009. Their mean age was 32.3 years. Among these patients, nineteen patients were ART-naïve (54.2%) and 16 were first-line ART failures (45.8%). In ART naïve patients, the median CD4 count was 144 cells/μl and the average viral load was 5.58 log10/ml (388 643 copies/ml). For the patients with first-line ART failure, the median CD4 count was 178 cells/μl, viral loads mean was 5.62 log10/ml and the median time of therapy was 30 months (Table 1). There were 20 females (57%) which were not pregnant at the time of blood collection. Genetic subtyping revealed that 65.7% of samples were infected with subtype C, 20% with CRF02_AG, 8.5% with B, 2.9% with CRF02_AG/C and 2.9% with K/C (Table 2).

**Table 1:** Patients characteristics

	**ART-naïve patients**	**Patients with first-line ART failure**
Effectives	19	16
Age (Mean, Years)	32	32.67
CD4 (cells/μl) Mean	144	178
CVp (log 10 copies/ml) Mean	5.58	5.62

**Table 2:** HIV-1 subtypes and distribution of resistance-associated mutations among 19 ART-naïve patients attending three health centers in Djibouti city between October and December 2009

**Patients**	**SEX**	**CD4**	**VL (cp/mL)**	**SUBTYPE**	**NRTI**	**NNRTI**	**Major Mutation PI**	**Minor Mutation PI**
1	M	208	170 000	C	None	None	None	None
2	M	107	4 690 000	C	None	None	None	None
3	F	186	1 600 000	CRFO2_AG	None	K103N	None	None
4	M	167	150 000	C	None	None	None	None
5	F	195	160 000	C	None	None	None	None
6	F	154	98 000	C	None	None	None	None
7	F	104	3 360	CRF02_AG	None	None	None	None
8	F	47	350 000	C	None	K101Q, K103N	None	None
9	M	126	76 000	CRFO2_AG	None	None	None	L10V
10	M	100	150 000	C	None	None	None	None
11	M	20	420 000	CRF02_AG	None	None	None	None
12	F	51	45 000	K/C	None	None	None	None
13	F	540	260 000	C	None	None	None	None
14	M	56	43 900	C	None	None	None	None
15	F	23	663 000	C	None	None	None	None
16	M	49	185 000	C	None	None	None	None
17	F	170	87 200	B	None	K101E	None	None
18	F	250	120 000	C	None	None	None	None
19	F	90	431 000	C	None	None	None	None

The ART naïve patients showed only NNRTI-associated mutations which were K103N (2 strains) and K101Q (1). No mutations of NRTI were detected. The minor PI mutation L10V was detected in one ART naïve patient [8].

At the time of sampling, all the patients receiving ART were treated by first line. According to the Djiboutian National Program for fighting against AIDS (PNLS) recommendations [9], the first-line treatment consists in 2 NRTI + 1NNRTI. The NRTI backbones were stavudine, zidovudine, and lamivudine. The most used drug combination (12 Patients (75%)) was AZT/3TC/EFV, while the 6 other patients received either AZT/3TC/NVP, D4T/3TC/EFV or D4T/3TC/NVP. The NRTI-associated mutations were D67N (2 strains), T69N (1), M184V (6), L210W (2), T215Y (2). The NNRTI-associated mutations were K101Q (1), K103N (4), V179E (1), Y181C (1), Y188L (2), G190A (2) (Table 3). For PI-associated mutations, the minor mutation L10V and other polymorphism were detected.

**Table 3:** HIV-1 subtypes and distribution of resistance-associated mutations among 16 patients with first-line ART failure attending three health centers in Djibouti city between October and December 2009

**N°**	**SEX**	**CD4**	**SUBTYPE**	**VL (cp/mL)**	**TREATMENT**	**NRTI**	**NNRTI**	**Major Mutation PI**	**Minor Mutation PI**
20	F	62	C	630 000	AZT/3TC/EFV	None	None	None	None
21	M	92	C	220 000	AZT/3TC/EFV	None	None	None	None
22	F	346	C	1 900	AZT/3TC/EFV	None	None	None	None
23	M	176	C	21 000	AZT/3TC/EFV	None	None	None	None
24	F	220	C	3270	AZT/3TC/EFV	None	None	None	None
25	F	243	C	7 500	AZT/3TC/EFV	None	None	None	None
26	F	103	C	413 000	D4T/3TC/NVP	None	K101Q, Y181C	None	None
27	M	52	C	26 000	AZT/3TC/EFV	M184V	K103N	None	None
28	M	249	C	213 000	D4T/3TC/EFV	D67N, M184V, L210W, T215Y	Y188L, G190A	None	None
29	F	230	C	1 080 000	AZT/3TC/EFV	M41L, D67N, L74V, M184V, L210W, T215Y	K103N, G190A, H221Y, M230L	None	None
30	F	140	CRFO2_AG	168 000	D4T/3TC/EFV	None	None	None	None
31	F	250	CRF02_AG/C	72 720	AZT/3TC/NVP	K70KR, M184V, K219R	K103N	None	None
32	M	92	CRFO2_AG	21 000	AZT/3TC/EFV	T69N	V179E, Y188L	None	L10V
33	M	88	CRFO2_AG	7 100	AZT/3TC/EFV	M184V	K103N	None	L10V
34	F	208	B	1 300	D4T/3TC/NVP	A62V	None	None	None
35	M	105	B	4 837 560	AZT/3TC/EFV	A62V, M184V, T215F, K219Q	None	None	None

Drug susceptibility results were classified into three categories: susceptible, intermediate resistance and resistance. Of the 16 patients with first-line ART failure, 56.25% showed resistance to at least one antiretroviral molecule: two patients (12.5%) were resistant to nucleoside (NRTI), one (6.25%) to non-nucleoside (NNRTI) reverse transcriptase inhibitors, and six (37.5%) to both. We found that the resistance rates to Lamivudine and Nevirapine in the 16 patients with first-line ART failure were 37.5% and 43.75% respectively. Forty four percent (44%) of sequences from viral strains did not show any mutation associated to ARV drug resistance- 7out of 16 [10]. Therefore, therapeutic solutions using second line drugs could be proposed to our patients with therapeutic failure and drug resistance mutations.

## Discussion

Minimizing drug resistance to highly active antiretroviral therapy (HAART) is important. First, it maximizes the opportunity for successful second-line and subsequent therapies after viral rebound during first-line treatment; second, it limits the transmission of drug-resistant viruses. We report results of the HIV-1 genetic diversity and prevalence of mutations associated with antiretroviral drugs among ART naïve as well as patients who failed first line therapy. The HAART was introduced in Djibouti in 2004. Djibouti, surrounded by Eritrea, Ethiopia, and Somalia, is an important crossroad for trade and exchange.

Our result revealed a majority of subtype C (~66%). In fact, Subtype C prevalence has been described in the Horn of Africa [11-14] and a similar prevalence was previously reported in Djibouti in 2005 [15,16]. However, our study describes the subtype B in Djibouti for the first time. It is the predominant subtype in the Western world and the clade that has circulated in North America and Europe. The detection of CRF02_AG strains indicates that they are still circulating in Djibouti, the only country in East Africa in which this recombinant virus was found some years ago [17,18]. CRF02_AG recombinant isolates were primarily described in West and Central Africa [19,20]. The discordances observed between RT and PR sequences in 2 isolates (CRF02_AG/C, K/C) are probably attributable to the high variability of the genome of HIV in particularly intra subtype recombination within group M subtype C [21].

For the ART naïve patients, the low rate of CD4 and the high rate of VL can be explain by the fact that 80% of patients infected by HIV in Djibouti was already detected at an advanced stage of the epidemic according to WHO classification [22,23]. Among the 19 strains, 2 were resistant only to non-nucleoside reverse transcriptase inhibitors (NNRTIs) while one strain showed a minor mutation to PI. Detection of RT and protease mutations associated with drug resistance in individuals who have not been exposed to antiretroviral therapy is thought to result from either transmission from a treated individual.

**Figure 1:**
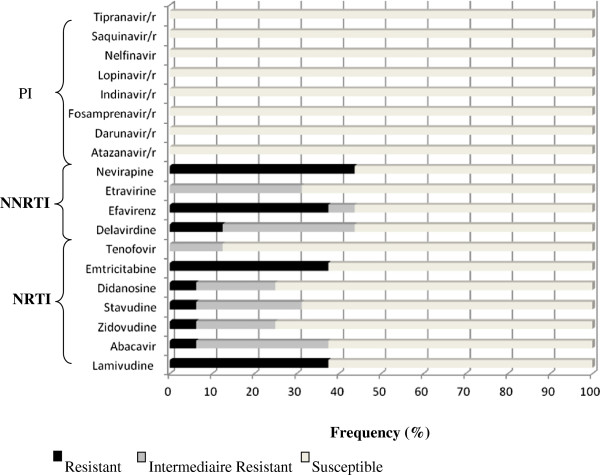
**Frequencies of resistance levels to nucleoside (NRTI) and non-nucleoside (NNRTI) reverse transcriptase inhibitors.** The horizontal bars indicates the frequency of susceptible (white), intermediate resistance (grey) and resistant (black) samples to NRTI and NNRTI reverse transcriptase inhibitors.

Concerning first-line ART failures, 38% of 16 strains sequenced in this group had the M184V mutation. This mutation is selected by lamivudine (3TC), emtricitabine (FTC) or abacavir (ABC) use and confers high-level resistance to 3TC (Figure 1) [24]. Patients receiving incompletely suppressive 3TC regimens usually develop M184V as their first mutation [25,26]. Accumulation of the other mutations observed included thymidine associated mutations (including M41L, D67N, K70R, L210W, T215Y/F, K219Q) results in increasing resistance to AZT, Tenofovir, D4T, Abacavir, and DDI [27,28]. Two multidrug resistance mutation profiles in general, confer high level drug resistance to all NRTIs. The most common occurring nucleotide excision mutations (NEMs) that confer resistance by enhanced excision [29] were at position 215. The T215Y mutation was observed in 13% of the sequences while T215F was detected in 6%. Clinical studies have shown that the NEMs, particularly mutations at position 215, interfere with the clinical response to zidovudine, stavudine, abacavir, didanosine, and most dual NRTI combinations [30]*.* Mutations conferring resistance to NNRTIs were also seen, with one strain showing resistance only to NNRTIs. The NNRTI resistance-conferring mutations included K103N (25%), Y188L (12.5%), Y181C (6%), G190A (6%), H221Y (6%), M230L (6%). Our data suggest the non-epidemic circulation of resistant viruses, and the absence of multi-class drug resistant viral strains.

It is important to appreciate that resistance at failure is only one factor to consider when choosing an initial HAART regimen. Coformulation, simplicity of administration, price, drug interactions (particularly with tuberculosis therapy), toxicity and adverse events are all important considerations and will differ between patient populations. We noticed seven patients (~44%) did not show resistance-conferring mutations and the therapeutic failure may have been due to other factors [31-35]. Information on treatment from their clinic folders allowed us to confirm that for five of these seven cases the ART failure profile was due to poor adherence and for the remaining two cases the detectable viral level was due to coinfection with tuberculosis and malnutrition [36-38].

The escalating rates of transmission of drug resistant virus observed in the past few years, coupled with the poorer response to treatment in persons with drug resistant virus, are being considered as a basis for a recommendation that resistance tests should be performed routinely for persons with new HIV infection [39]. This evaluation, both at the baseline and in follow-up, will be crucial to assess the impact of therapy regimens on resistance development and also their adequation.

## Conclusion

This study reveals primary mutations that confer resistance to NRTI, NNRTI in HIV-1 non-B and B subtype strains, isolated from ART naïve as well as patients who failed first line therapy in Djibouti. It also illustrates the importance of preliminary surveys of drug-resistance mutations in resource-poor countries. Therefore, we suggest the use of resistance testing to check the prevalence of the drug resistance mutation that arises following failure of the first line regimen. This will help in establishing guidelines for second line regimens in Djibouti. This allows the selection of adapted antiretroviral regimens in these regions. Our results provide a basis for repeated epidemiological studies to measure the population effects of HIV treatment programs over time.

## Abbreviations

ART: Antiretroviral therapy; PR: Protease; RT: Reverse Transcriptase; ARV: Antiretroviral.

## Competing interests

The authors declare that they have no competing interests.

## Authors’ contributions

AEA: wrote the manuscript, has made substantial contributions to acquisition of data, or analysis and interpretation of data. AEA and AJ: participated in data analysis and interpretation. HYD and MA BHK: revised the manuscript. AS: proposed the idea, revised the manuscript and save final approval of the version to be published. All authors read and approved the final manuscript.
